# More men than women make mucosal IgA antibodies to Human papillomavirus type 16 (HPV-16) and HPV-18: a study of oral HPV and oral HPV antibodies in a normal healthy population

**DOI:** 10.1186/1471-2334-6-95

**Published:** 2006-06-08

**Authors:** Dianne J Marais, Candice Sampson, Anthea Jeftha, Dherendra Dhaya, Jo-Ann S Passmore, Lynette Denny, Edward P Rybicki, Eric Van Der Walt, Lawrence XG Stephen, Anna-Lise Williamson

**Affiliations:** 1 Institute of Infectious Disease and Molecular Medicine, Faculty of Health Sciences, University of Cape Town, Observatory, 7925, Cape Town, South Africa; 2 Departments of Oral Medicine & Periodontology, Faculty of Dentistry, University of the Western Cape, Cape Town, South Africa; 3 National Health Laboratory Services, Groote Schuur Hospital, Observatory, 7925, Cape Town, South Africa; 4 Department of Molecular and Cell Biology, University of Cape Town, Rondebosch, 7701, Cape Town, South Africa; 5 Department of Obstetrics and Gynaecology, Groote Schuur Hospital, Observatory, 7925, Cape Town, South Africa

## Abstract

**Background:**

We have previously shown the high prevalence of oral anti-human papillomavirus type 16 (HPV-16) antibodies in women with HPV-associated cervical neoplasia. It was postulated that the HPV antibodies were initiated after HPV antigenic stimulation at the cervix via the common mucosal immune system. The present study aimed to further evaluate the effectiveness of oral fluid testing for detecting the mucosal humoral response to HPV infection and to advance our limited understanding of the immune response to HPV.

**Methods:**

The prevalence of oral HPV infection and oral antibodies to HPV types 16, 18 and 11 was determined in a normal, healthy population of children, adolescents and adults, both male and female, attending a dental clinic. HPV types in buccal cells were determined by DNA sequencing. Oral fluid was collected from the gingival crevice of the mouth by the OraSure method. HPV-16, HPV-18 and HPV-11 antibodies in oral fluid were detected by virus-like particle-based enzyme-linked immunosorbent assay. As a reference group 44 women with cervical neoplasia were included in the study.

**Results:**

Oral HPV infection was highest in children (9/114, 7.9%), followed by adolescents (4/78, 5.1%), and lowest in normal adults (4/116, 3.5%). The predominant HPV type found was HPV-13 (7/22, 31.8%) followed by HPV-32 (5/22, 22.7%). The prevalence of oral antibodies to HPV-16, HPV-18 and HPV-11 was low in children and increased substantially in adolescents and normal adults. Oral HPV-16 IgA was significantly more prevalent in women with cervical neoplasia (30/44, 68.2%) than the women from the dental clinic (18/69, 26.1% P = 0.0001). Significantly more adult men than women displayed oral HPV-16 IgA (30/47 compared with 18/69, OR 5.0, 95% CI 2.09–12.1, P < 0.001) and HPV-18 IgA (17/47 compared with 13/69, OR 2.4, 95% CI 0.97–6.2, P = 0.04).

**Conclusion:**

The increased prevalence of oral HPV antibodies in adolescent individuals compared with children was attributed to the onset of sexual activity. The increased prevalence of oral anti-HPV IgA in men compared with women was noteworthy considering reportedly fewer men than women make serum antibodies, and warrants further investigation.

## Background

The involvement of human papillomaviruses (HPV) in squamous cell carcinomas of the anogenital region is widely accepted. HPV infection has also been demonstrated in several disorders of the oral and tonsillar regions [1] but unlike cervical cancers where almost 100% of tumours contain HPV DNA [2], only up to half of oral and tonsillar cancers contain HPV DNA, the greater majority with HPV types HPV-16 and HPV-18 [1]. HPV has been reported present in normal buccal mucosa with varying detection rates [3-5]. Oral HPV infection shows the typical fluctuating presence observed in anogenital mucosa [6].

Vaccines for the control of HPV infection are presently in the process of being released for general use. In Africa with its huge burden of HPV-associated cancers, novel vaccines against HPV are under development that could enable the vaccination of large sectors of the population [7]. The introduction of appropriate vaccines to an area will require knowledge of the HPV types within the general population and those associated with cervical [8] and other cancers. Vaccine introduction will also require monitoring of the immune response in vaccinees during clinical trials and then within a public health vaccine program the testing of children and young people for exposure to HPV prior to vaccination. Therefore, there is the need for easy, safe, non-invasive sampling methods for the determination of HPV infection and of the immune responses to HPV.

The testing of oral fluid for antibodies has proved most useful as an HIV-1 screening tool as oral HIV-1 IgG antibodies closely reflect HIV-1 serostatus [9]. The oral test requires the insertion of a small absorbent pad into the gingival crevice of the mouth for two minutes. Using this sampling method, we previously described the presence of oral fluid HPV-16 IgA and IgG antibodies in the majority of women with cervical neoplasia [10]. In a small pilot study we found that oral HPV-16 IgA, when compared with serum and cervico-vaginal rinse antibodies, most closely correlated with HPV-16 DNA at the cervical lesion of women with cervical intraepithelial neoplasia (CIN) [7] This indicated that oral IgA could be a useful biomarker of mucosal HPV infection at a genital site via the common mucosal immune system [11]. Cameron et al., 2003 [12] reported a moderate correlation between oral and serum HPV IgG antibodies in HIV-1 seropositive individuals. Buchinsky et al., 2006 [13] aiming to evaluate oral fluid testing in lieu of serum testing for HPV antibody status, reported a concordance of oral fluid and serum antibodies from college students but that oral antibody detection was less sensitive than serum.

HPV seropositivity has been shown to be a biomarker of past and present HPV infection and lifetime number of sexual partners [14]. The presence of oral fluid HPV IgG being mainly serum-derived [15] could conceivably serve as a similar biomarker.

The present study aimed to evaluate the extent of oral HPV infection and the prevalence of oral IgA and IgG antibodies to HPV-16, HPV-18 and HPV-11 in members of a normal healthy population, male and female, children to adult, and to assess the efficacy and acceptability of oral fluid sampling for HPV testing in young children and older individuals. To our knowledge the presence of HPV antibodies in oral fluid have not previously been reported in such populations. The study also hoped to further our limited basic understanding of the immune response to HPV infection in especially men who are being considered for inclusion in HPV vaccination strategies as men sexually transmit HPV to their female partners[14]. Significantly fewer men reportedly make serum HPV antibodies than women [16-18] despite relatively higher rates of sexual activity [16].

## Methods

### Study participants

Participants in the present study were visitors to dental clinics in the Cape Town metropolitan area for normal dental treatment during 2003(dental clinic population). Informed consent or parental consent for the children was obtained from all individuals and approval for the study obtained from the Ethics Committee for Human Research of the University of the Western Cape. There were 114 children (56 male, 58 female, aged 1–12 years, mean age 6 years), 78 adolescents (36 male, 42 female, aged 13–19 years, mean age 15.7 years) and 116 adults (47 male, 69 female, aged 20–61 years, mean age 28.8 years) with adequate buccal samples who were included in the study. Further participants were 44 women who formed part of study investigating cervical immune responses to HPV-16 (Passmore et al., submitted for publication). The latter women aged between 18 and 40 years were recruited during 2004 and 2005 from amongst first time attendees of the Colposcopy Clinic at Groote Schuur Hospital, Cape Town, South Africa. Of the 44 women, 19 were diagnosed by histology and/or colposcopy with high grade cervical intraepithelial neoplasia (CIN2/3), 13 with low grade cervical disease (CIN1) and 12 had cleared their CIN (CIN0) (colposcopy clinic, CC patients). The latter (CIN0) had been referred to the colposcopy clinic with high grade cervical disease as diagnosed by Pap smear but on subsequent histology or colposcopy or both showed no CIN.

### Sample collection

Trained dentists collected buccal samples for HPV DNA determinations by brushing the entire mucosa of both inner cheeks with a CerviBrush (Digene). Brushes were placed in sterile Digene tubes containing transport buffer. Oral fluid samples were collected with an OraSure pad (Epitope, Beaverton, OR) as described previously [10], sampling predominately serum transudate in the gingival crevice of the mouth. Oral fluid IgA collected by this method is mainly saliva derived whereas oral IgG is primarily from serum transudate [15]. The presence of any oral lesion in the dental population was diagnosed by consensus of at least two consultant dentists. The presence of gingivitis was not assessed in any patient. From the CC patients enrolled in the study, a Digene cervical sampler obtained cervical cells and mucus from the endocervix for HPV DNA and antibody determination and clotted peripheral blood specimens were taken for the measurement of serum antibody responses.

### HPV DNA determinations

DNA was extracted and purified from buccal cells using QIAamp DNA minikit (Qiagen) and buccal swab protocol. The quality of the DNA and thus the adequacy of the buccal sample, was evaluated by amplifying a section of the CCR-5 gene as described by Michael et al, (1997) [19]. The presence of oral HPV DNA in the dental population was detected using degenerate primers [20] in a nested polymerase chain reaction producing a 335 bp fragment that was purified and sequenced. HPV typing was performed on cervical cells from CC patients using a Roche Reverse line blot hybridization assay according to the manufacturers' instructions (Roche, USA). The Roche reverse line blot hybridization assay has the capacity to detect 37 different HPV genotypes (Oncogenic types: HPV-16, -18, -26, -31, -33, -35, -39, -45, -51, -52, -53, -56, -58, -59, -66, -67, -68, -69, -70, -73, -82, -IS39 [n = 22]; nononcogenic types: HPV-6, -11, -40, -42, -54, -55, -61, -62, -64, -71, -72, -81, -83, -84, -CP6108 [n = 15]).

### Virus-like particles

All virus-like particles (VLPs) for antibody enzyme-linked immunosorbent assay (ELISA) were composed of major capsid protein L1 only. HPV-18 VLPs were obtained from MedImmune Inc (Gaithersburg, USA). HPV-16 and HPV-11 VLPs were prepared using recombinant baculoviruses expressing either HPV-11 or HPV-16 L1 protein to infect monolayer cultures of Sf21 insect cells in twelve 75 cm^2 ^tissue culture flasks, at an MOI of ~10. Cells were harvested by centrifugation at 5000 × g, 6 days post infection [21]. The cell pellet was resuspended in 10 ml harvest buffer (Dulbecco's PBS, Sigma, plus 0.4 g/ml CsCl) and the cells lysed by sonication. The cell lysate was subjected to isopycnic gradient ultracentrifugation at 100 000 × g for 24 hours at 20°C using a Beckman SW50.1 rotor. The VLP-containing band was removed through the side of each tube using a hypodermic needle and syringe. The VLP preparations were then dialysed extensively against dialysis buffer (PBS plus 400 mM NaCl) before being frozen in aliquots at -80°C until use.

### Antibody determination

A capture ELISA was used to detect oral HPV IgA and IgG antibodies to HPV-16, HPV-18 and HPV-11. Oral fluid and serum from CC patients were tested for HPV-16 antibodies only. Serum antibody detection was by direct VLP-16-based ELISA. ELISAs were a modification of that described by Studentsov et al., 2002 [22], using polymer solutions (Sigma) for blocking (polyvinyl alcohol, PVA) and secondary antibody (polyvinylpyrrolidone, PVP) enhancement. This enhanced ELISA enabled the use of oral fluid at a 1:5 dilution instead of the previous 1:1 dilution [10] and serum at 1:100 dilution. Briefly, monoclonal antibodies H16.V5 directed against the major conformational, neutralising epitope on HPV-16, H11 B3 against HPV-11 and H18 R5 against HPV-18 (kindly supplied by N. Christensen, Milton Hershey Medical Centre, USA) were used as capture antibodies. Plates were coated with 100 μl monoclonal antibody at a dilution of 1:2000 in PBS pH 7.4 overnight at 4°C, washed twice with PBS and blocked with 100 μl 0.05% PVA in PBS for 2 hours at room temperature. Plates were washed six times with PBS and wells filled with 100 μl of 0.05 μg virus-like particle (VLP) to HPV-16, HPV-11 and HPV-18 in PBS for 1 hr at 37°. After washing plates six times, oral or cervical fluid diluted 1:5 in 0.5% PVA was added and allowed to incubate at 40°C overnight. Subsequent procedures were as described for other ELISAs [10] but using 0.8% PVP and 0.5% PVA in PBS for the dilution of the horseradish peroxidase conjugated anti-human IgA and IgG and six washes between ELISA procedures. The cut-off for oral antibody positivity was determined by calculating the mean plus 3 standard deviations of the absorbance values obtained from the oral fluid samples of those children aged between 2 and 10 years after the elimination of outliers as described for the determination of seropositivity cut-off values [23]. Total IgA estimations were performed on each oral sample to determine the adequacy of the sample prior to HPV antibody testing, as described previously[10]. Total oral IgA mean values were 2.3 μg/ml in children, 4.7 μg/ml in adolescents and 1.8 μg/ml in adults. There were 3 samples from children with below normal levels of oral fluid total IgA (<0.5 μg/ml) [24] and these children were not included in the study. To determine the presence of HIV-1 infection, oral samples were tested was by GACELISA (Wellcozyme HIV-1 + 2, Murex). HIV-1 testing was anonymous and unlinked so no HIV-1 counselling was done. HIV-1 infection was found in 1/114 children, 1/78 adolescents and 0/116 adults. CC patients were not tested for HIV-1 seropositivity.

### Statistical analysis

The X^2 ^test of independent proportions was used to determine the Odds Ratios (OR) and 95% confidence intervals (95% CI) for the association of antibodies between different groups. Agreement between IgA and IgG within the same sample and between serum and oral fluid antibodies within the same individual was evaluated by Kappa statistic. If Kappa = 1, then there is perfect agreement and if Kappa = 0 there is no agreement. A kappa <0.4 indicates that there is poor agreement, a kappa 0.4–0.75 indicates fair to good agreement and a kappa > 0.75 indicates excellent agreement. [25].

## Results

### Oral HPV DNA

All CerviBrush-collected buccal samples were CCR-5 positive. Extraction controls (phosphate buffered saline, PBS) were included during all extractions and all were negative for both CCR-5 and HPV DNA. Oral HPV DNA was detected in 17/308 buccal specimens from the dental population (5.5%) with the prevalence of oral HPV highest amongst the children (9/114, 7.9%) then the adolescents (4/78, 5.1%) and lowest amongst adults (4/116, 3.5%). The most prevalent type was HPV-13 found in 41.2% (7/17) of the HPV positive samples or in 2.3% of the participants. The demographics, oral HPV types and oral HPV-16, HPV-18 and HPV-11 antibody prevalence of the 17 people infected with oral HPV are shown in Table 1. The one patient diagnosed with focal epithelial hyperplasia was infected with HPV-13 (Table 1). There was no apparent gender difference in oral HPV prevalence (P = 0.7). There were 9 male and 8 females of the 17 individuals infected with oral HPV (Table 1)

**Table 1:** Age, gender and prevalence of oral HPV types and oral HPV antibodies in the 17 dental patients with oral HPV infection

**Patient count**	**Age years**	**Gender**		**Oral HPV type**	**Oral lesions**	**VLP-16**		**VLP-18**		**VLP-11**	
		**Male**	**Female**			**IgA**	**IgG**	**IgA**	**IgG**	**IgA**	**IgG**
1	4	+		13							
2	5	+		13							
3	6	+		32							
4	6		+	32							
5	7	+		11							
6	7		+	32							
7	8	+		72		+					
8	9		+	72							
9	10	+		13							
10	13		+	32							
11	15	+		31				+			
12	15		+	11							
13	15		+	13							
14	22		+	13	FEH						
15	23		+	16			+				
16	48	+		13		+					
17	52	+		13		+	+				+

• FEH = Focal epithelial hyperplasia

### Oral antibodies in children, adolescents and adults

**Figure 1:**
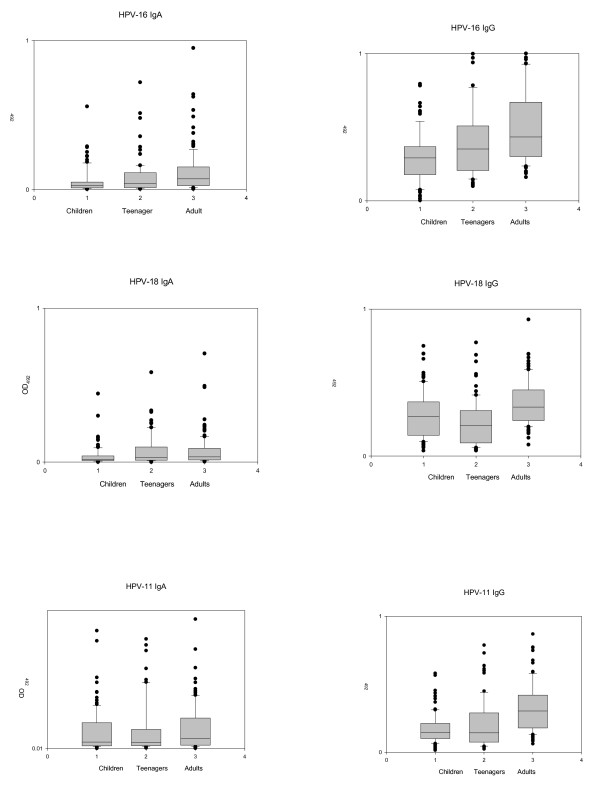
**Boxplot analysis of dental patients HPV ELISA OD results**. Box plot analysis of the optical density values obtained in oral antibody ELISA to HPV-16, HV-18 and HPV-11 in dental clinic patients, children, adolescents and adults. Median values, depicted by the line in the box, for HPV-16 IgA are 0.03, 0.04 and 0.08 for children, adolescents and adults respectively: for HPV-16 IgG 0.29, 0.35, and 0.43 respectively: for HPV-18 IgA 0.02, 0.03 and 0.03 respectively and HPV-18 IgG 0.3, 0.2 and 0.4 respectively: for HPV-11 IgA 0.02, 0.02, and 0.04 and HPV-11 IgA 0.15, 0.14 and 0.31 respectively

**Figure 2:**
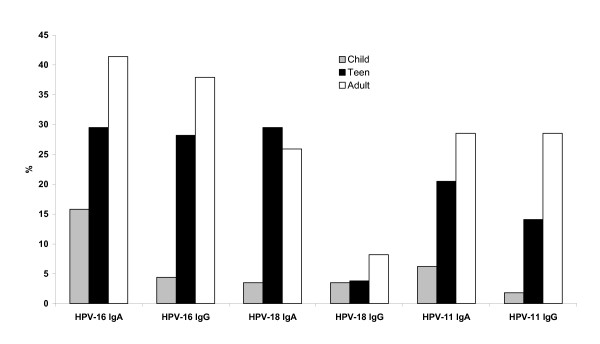
**HPV antibody prevalence in the dental cohort**. The prevalence of HPV-16, HPV-18 and HPV-11 IgA and IgG antibodies in dental clinic patients, children (child), adolescents (teen) and adults.

In contrast to the oral HPV prevalence, the prevalence of oral HPV antibodies was lowest in the children, increased in adolescents and was highest in adults (Fig 1). Of the children 15.8% (18/114) and 4.4% (5/114) tested positive for oral HPV-16 IgA and IgG respectively. For HPV-18 the prevalence was 3.5% (4/114) for both IgA and IgG and for HPV-11, 6.2% (7/114) and 1.8% (2/114) respectively. Oral antibody prevalence increased in adolescents where 29.5% (23/78) and 28% (22/78) displayed HPV-16 IgA and IgG respectively, 29.5% (23/78) and 3.8% (3/78) HPV-18 IgA and IgG and 21% (16/78) and 14.1% (11/78) HPV-11 IgA and IgG respectively. The difference in antibody prevalence between children and adolescents were significant for HPV-16 IgA (P = 0.01) and IgG (P < 0.001), for HPV-18 IgA (P = 0.001) and for HPV-11 IgA (P = 0.002) and IgG (P = 0.001). The highest antibody prevalence was seen in the adults where 41% (48/116) and 37.9% (44/116) showed HPV-16 IgA and IgG, 25.9% (30/116) and 8.2% (9/116) HPV-18 IgA and IgG respectively and 28.5% (33/116) for HPV-11 IgA and IgG (Fig 2). Differences were not significant between adult and adolescent antibody prevalence except for HPV-11 IgG (P = 0.02). Other P values were, HPV-16 IgA P = 0.9, HPV-16 IgG P = 0.16, HPV-18 IgA P = 0.6 and HPV-18 IgG P = 0.4 and HPV-11 IgA P = 0.5. Differences between adult and children antibody prevalences were all highly significant (P < 0.001) except for HPV-18 IgG (P = 0.15).

There was low concordance between the prevalence of oral HPV-16, HPV-18 or HPV-11 IgA and IgG antibodies within the same sample as determined by kappa (K < 0.4, data not shown). There were 15/116 (12.9%) adults with corresponding IgA and IgG to HPV-16, three of these adults also had corresponding IgA and IgG to HPV-18 and to HPV-11 and 3 had corresponding IgA to HPV-11 as well as HPV-16. No adult had corresponding IgA and IgG to HPV-18 or HPV-11 unless they had corresponding HPV-16 IgA and IgG antibodies (data not shown).

### Oral antibodies stratified by gender

**Figure 3:**
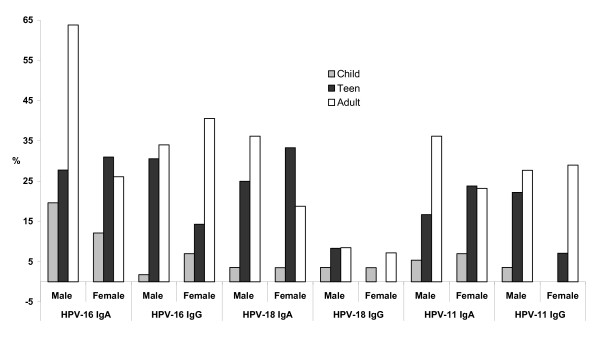
**Antibody prevalence in dental cohort according to gender**. The prevalence of oral HPV-16, HPV-18 and HPV-11 antibodies in dental clinic patients, children (child), adolescents (teen) and adults according to gender.

Antibody prevalence in oral fluid was assessed according to gender in the three age categories of dental clinic participants (Fig 3). Amongst the adults more men displayed HPV-16 and HPV-18 and HPV-11 IgA than females (64% compared with 26%; 36% compared with 19% and 36% compared with 23% respectively). The male to female differences were significant for HPV-16 IgA (30/47 compared with 18/69, OR 5, 95% CI 2.1–12.1, P < 0.001) and borderline for HPV-18 IgA (17/47 compared with 13/69, OR 2.4 95% CI 0.97–6.2, P = 0.04) but not significant for HPV-11 (17/47 compared with 16/69, OR 1.9, 95% CI 0.8–4.6, P = 0.13). The prevalence of HPV IgG was similar in men and women (34% compared with 41% for HPV-16; 8.5% compared with 7.2% for HPV-18 and 28% compared with 29% for HPV-11 (P = 0.5, P = 0.5 and P = 0.9 respectively) (Fig 2). The number of oral antibody positive children was too low for a gender comparison to be made except for HPV-16 IgA where the male to female difference was not significant (11/56 compared with 7/58, OR 1.63 95% CI 0.6-7-3.9, P = 0.3). For the adolescents the trend was for more females to display oral HPV IgA and more males HPV IgG, although differences were not significant. Oral HPV-16 IgA was found in 31% of female adolescents and in 27.8% of adolescent men (P = 0.76). For HPV-18 IgA the prevalences were 33% and 25% (P = 0.42) and HPV-11 IgA 24% and 17% (P = 0.43) for young women and young men respectively (Fig 3). An apparent increased prevalence of oral HPV-16, HPV-18 and HPV-11 IgA in female adolescents compared with adult females was also not significant (Fig 3). Amongst adolescent females the prevalence of HPV-16, HPV-18 and HPV-11 IgA was 31%, 33.3% and 24% respectively and for adult females these prevalences were 26%, 18.8% and 23.2% respectively (P = 0.6, 0.08 and 0.9 respectively).

### Antibodies in women from the colposcopy clinic

Amongst the women from the colposcopy clinic, HPV-16 oral IgA was found in 30/44 women (68.2%) and HPV-16 oral IgG in 14/44 (31.8%) women. The prevalence of oral HPV-16 IgA was significantly higher in these women than the women from the dental clinic (30/44, 68.2% compared with18/69, 26.1% P = 0.0001) but not for oral HPV-16 IgG (14/44 31.8%, compared with 28/69, 40.6% P = 0.35). The prevalence of HPV-16 serum IgA in the colposcopy clinic patients was 50%, serum IgG 34.1%. There was low agreement between the presence of oral or serum HPV-16 antibodies in the same individual. There was also low agreement between the presence of HPV-16 IgA or IgG within the same individual (Kappa < 0.4, data not shown).

The prevalence of HPV-16 antibodies in oral and serum samples from these women stratified according to the grade of CIN and the presence of cervical HPV-16 are shown in Table 2. There were no significant differences between the prevalence of antibodies in oral or serum samples from women with cervical HPV-16 infection and those without. Oral HPV-16 IgA antibody prevalence was 52.6% in women with HPV-16 cervical infection and 68.2% in women with no HPV-16 infection (P = 0.09) and oral HPV-16 IgG 29.4% and 33.3 % respectively (P = 0.79). For serum antibodies, HPV-16 IgA was found in 35.3% of HPV-16 infected women and in 50% of women with no HPV-16 (P = 0.12) and HPV-16 IgG found in 29.4% and 34.1% (P = 0.60).

**Table 2:** The prevalence of HPV-16 antibodies in CIN patients (Pt) according to their grade of CIN (CIN0, CIN1 or CIN2/3) and HPV-16 infection at the cervix.

	Pt	HPV types	HPV-16 IgA		HPV-16 IgG	
	no	cervical	Oral	serum	oral	serum

CIN 0	88	11, 16, 18, 35, 42, 55				
	79	16, 18, 31, 35, 55, 58	■		■	■
	87	16, 51, 55			■	
CIN 1	92	16	■		■	
	85	16, 58, 61	■	■		■
CIN 2/3	74	16				
	80	16				
	86	16	■			
	93	16				
	95	16	■			
	122	16	■	■		■
	123	16, 45, 70, 61		■		■
	108	16, 45, 70, CP6108		■		
	109	16, 51, IS39, 83		■		■
	121	16, 68	■	■		
	99	16, 39	■		■	
	100	16, 52, 66	■		■	
Number	17		9	6	5	5
%			52.9	35.3	29.4	29.4
CIN 0	103	35	■	■		■
	111	53	■	■		
	112	58	■		■	
	90	33, 61	■	■	■	
	75	42, 51, 56		■		
	104	51, 56				■
	107	negative	■	■		
	110	negative	■	■		■
	120	negative	■	■		
CIN 1	119	45	■	■		
	101	51	■	■	■	■
	106	53	■	■		
	84	56	■			■
	78	35, 59				
	89	35, 62				
	91	51, 53, 54, 59, 68	■		■	■
	116	52, 33, 35, 58	■	■	■	■
	115	58, 66		■		■
	76	negative	■		■	
	114	negative	■	■		
CIN 2/3	117	35		■		
	105	18, 6	■			
	113	31, 45	■	■		
	94	31, 58	■		■	
	118	33, 45, 58, 66	■	■		■
	82	45, 82	■		■	
	81	51, 58	■		■	■
Number	27		21	16	9	10
%			77.8	59.3	33.3	37.0

total	44		68.2	50.0	31.8	34.1

Empty cells indicate a negative antibody result

Black boxes indicate a positive antibody result

## Discussion

The oral HPV types found in this study belong to the *Alpha-papillomavirus *genus which includes mucosal and cutaneous HPV types [26]. The predominant type found was HPV-13, in 41.2% of the HPV positive isolates from the dental population. Focal epithelial hyperplasia (FEH) has been associated with both HPV-13 and HPV-32 [27]. HPV-13 was isolated from the mouth of the one individual with FEH lesions, supporting this association. The 5.5% (17/308) oral HPV prevalence found in the normal buccal mucosa of the dental clinic participants is similar to other studies [3,4,28]. The high prevalence of oral HPV in children has also been described [29] and was not reflected by a high oral antibody prevalence indicating that oral antibodies are possibly elicited elsewhere and not in the mouth. It must be noted however that we did not test for antibodies to the majority of the HPV types found in the mouth. The high prevalence of oral IgA in the women with CIN and especially in those without cervical HPV-16 infection is unclear. The apparent lack of concordance between oral and serum antibodies in the women with CIN confirms previously published data [10] and possibly indicates the distinct compartmentalisation of these responses.

The oral sampling of oral fluid for this study was favourably accepted by the children, adolescents and adults, even in children as young as one year old. Three oral fluid samples were discarded because they showed a lower than normal total IgA level (<0.5 μg/ml). This occurred in children < 2 years and was probably a consequence of the sampling device not remaining in the mouth for the correct length of time. A significant finding was the increased prevalence of oral HPV IgA in adult men compared with women. Gender differences in seropositivity to HPV have been reported. The prevalence of HPV serum antibodies is apparently consistently lower in men than in women, even in men with high risk sexual activity [16,18]. The reason for this has not been elucidated. HPV infection has been shown to play an important role in all anogenital cancers with HPV seropositivity in men and women varying according to the site of HPV infection [30]. The site of HPV infection could also impact on the HPV oral humoral responses, both IgA and IgG. Oral IgG in the present study originated to a certain degree from oral mucosal transudate and would reflect serum IgG antibodies [9] but has also been shown to be locally produced [24]. The significant number of women with cervical disease displaying oral anti-VLP-16 IgA supported a previous study which suggested that the IgA antibodies arose through the CMIS as a result of antigenic stimulation at a cervical site [10].

We postulate the site of the HPV infection in the men (and women) with oral antibodies to be anogenital, based on our findings in women with cervical neoplasia. In support of this, almost no HPV-16, HPV-18 and HPV-11 types were found in the mouth. The role of HPV infection in cancers at male and female anogenital sites has been clearly demonstrated [30]. Tonsillar HPV infection and oral sex have also been shown to be associated with HPV seroreactivity in men [18] so the tonsils could be the site of some of the HPV infection in the men in the present study. The distinct gender differences both in oral and serum HPV IgA and IgG antibodies require elucidation. The difference is possibly a consequence both of the site of infection and the type and extent of immune response that is elicited. IgG antibodies in the gingival crevice of the mouth are mainly serum derived and oral IgA locally produced so the differences could reflect differences in the humoral responses in systemic and mucosal compartments in men and women.

In the present study, the high prevalence of oral HPV antibodies in adolescents was notable. HPV seropositivity is related to sexual activity [14] and oral IgG sampled by the OraSure method, as in the present study, is largely serum derived. So if the postulated anogenital site of the initiation of oral IgA antibodies is correct, the appearance of oral HPV antibody probably correlated with the onset of sexual activity in the adolescents. The low prevalence of oral fluid HPV antibodies in children correlates with the low prevalence found in children's sera and substantiated the premise that oral antibodies are sexually associated as are serum antibodies.

## Conclusion

We believe that sampling oral fluid for HPV antibodies shows promise as a simple, non-invasive method to test for the presence of antibody responses to HPV infection. However larger studies are required to validate oral fluid antibody sampling as either a surrogate for serum testing or as a biomarker of anogenital HPV infection. We postulate the site of infection eliciting oral HPV antibodies to be mainly anogenital. As an adjunct to serology, oral testing could increase the sensitivity of HPV analysis, covering a broader range of humoral responses and contribute to our understanding of the immune response to HPV. It is well known that HPV serology underestimates genital HPV infection even when restricted to the same type HPV DNA as detected in the lesion. Conceivably certain individuals could mount more effective mucosal responses to HPV infection than systemic responses and oral IgA testing could serve as an adjunct to or even replace serum testing for genital HPV infection in children. A large study is underway to further investigate oral HPV IgA as a biomarker of genital HPV infection in women.

Most significant was the finding that more men than women display oral antibodies, considering the opposite is found for serum responses. Studies are planned to assist in the further understanding of the mucosal immune response to HPV in men and the site of the HPV infection eliciting these responses. The possible association of oral HPV antibodies with genital HPV infection especially in adolescents requires further clarification.

## Competing interests

The author(s) declare that they have no competing interests.

## Authors' contributions

DM: manuscript writing, antibody determinations, data analysis and data interpretation; CS: Buccal HPV type determination; AJ and DD: acquired dental patient data and collected the buccal samples; LS: participated in study design and coordination; JP designed and co-ordinated the study of the immune responses in women with CIN; LD provided the samples from the women attending the colposcopy clinic; EPR and EvdW: manuscript review and development and production of HPV-16 and HPV-11 virus-like particles; A-LW: Principal Investigator on the study – participated in the study design, manuscript review. All authors read and approved the final manuscript.

## Pre-publication history

The pre-publication history for this paper can be accessed here:


